# An Interferon-Response Transcriptomic Signature of Lymphovascular Invasion in Prostate Cancer

**DOI:** 10.3390/ijms27052167

**Published:** 2026-02-25

**Authors:** Cagdas Aktan, Christina M. Breneman, Okan Argun, Nora Seeley, Ceren Atalar, Kendall Robinson, Ari S. Hilibrand, Sophia Li, Swati Mamidanna, Mutlay Sayan

**Affiliations:** 1 Department of Medical Biology, Faculty of Medicine, Bandirma Onyedi Eylul University, Balikesir 10250, Türkiye; 2 Department of Radiation Oncology, Brigham and Women’s Hospital and Dana-Farber Cancer Institute, Harvard Medical School, Boston, MA 02115, USA; 3 Department of Radiation Oncology, Rutgers Cancer Institute of New Jersey, Rutgers Robert Wood Johnson Medical School, New Brunswick, NJ 08901, USA

**Keywords:** prostate cancer, lymphovascular invasion, transcriptomic

## Abstract

Lymphovascular invasion is an adverse pathologic feature in prostate cancer, but its independent molecular drivers remain unclear due to strong confounding by tumor grade and stage. We performed a confounder-adjusted transcriptomic analysis of 403 TCGA-PRAD samples. Differential expression was adjusted for Gleason score and pathological T stage. A transcriptional profile associated with LVI was derived and tested in multivariable logistic and Cox proportional hazards models for biochemical recurrence-free survival, with bootstrap internal validation. After multivariable adjustment, 129 genes were independently associated with LVI. This gene set was overwhelmingly enriched for interferon-alpha/beta signaling and antiviral response pathways. A continuous composite score derived from this profile predicted a reduced risk of biochemical recurrence independently of standard clinicopathological factors (adjusted HR per unit = 0.911, 95% CI: 0.835–0.993, *p* = 0.033). Multi-omics integration revealed subtle promoter hypomethylation and strong correlations between methylation and expression for key interferon genes, supporting transcriptional regulation. We identify a robust, interferon-response transcriptional profile that specifically defines LVI in prostate cancer after accounting for major clinical confounders. This transcriptional signature provides independent prognostic information, refines the biological understanding of LVI, and presents a novel targetable pathway for further investigation.

## 1. Introduction

Lymphovascular invasion (LVI) is a well-recognized histopathologic feature observed across a wide range of solid malignancies. In prostate cancer, the second most diagnosed cancer among men worldwide, LVI has emerged as a promising biomarker associated with adverse pathological characteristics and unfavorable clinical outcomes. LVI is defined as the presence of tumor cells within the lumen of lymphatic or blood vessels, enabling the tumor to breach vascular barriers and access systemic circulation. It is observed in a substantial proportion of prostate cancer cases, particularly those with higher Gleason grades and advanced-stage disease [1,2]. As an established risk factor for nodal metastasis, LVI represents a critical step in the metastatic cascade, facilitating the spread of cancer cells to regional lymph nodes and distant sites [2,3,4,5]. Given that approximately 40% of patients will experience biochemical recurrence following prostatectomy, identifying predictors of recurrence and overall risk remains essential for improving outcomes [6,7].

Clinically, LVI is strongly associated with adverse pathological features, including higher Gleason score, advanced tumor stage, extraprostatic extension, seminal vesicle invasion, positive surgical margins, cribriform architecture, and lymph node metastasis [8,9,10,11,12,13]. Collectively, these observations establish LVI as a robust marker of aggressive disease biology and adverse oncologic outcomes following radical prostatectomy, positioning it as a clinically relevant factor for postoperative risk stratification. Numerous retrospective and meta-analytic studies have shown that LVI is an independent predictor of biochemical recurrence, metastatic progression, and reduced overall survival following radical prostatectomy, further strengthening the implications of LVI as a predictor of prostate cancer prognosis [14,15,16,17]. The presence of LVI confers a risk profile similar to, or synergistic with, lymph node involvement, and may serve as a less invasive surrogate to inform decisions regarding adjuvant therapy [12,16].

Despite its recognized clinical and prognostic importance, a critical knowledge gap remains that has limited the broader clinical application of LVI. To date, molecular studies of LVI in prostate cancer have largely relied on unadjusted comparisons, making it difficult to distinguish LVI-specific biology from transcriptional programs driven by tumor grade, pathological stage, or tumor microenvironment composition [18,19]. As a result, the molecular underpinnings of LVI are poorly defined and remain confounded by its strong association with high-grade and high-stage disease. While transcriptomic profiles associated with LVI have been reported in other malignancies, this has not been systematically established in prostate cancer [20,21]. It therefore remains unclear whether LVI harbors an independent molecular profile or merely reflects the transcriptional profile of aggressive tumors in general. Disentangling this confounding is essential to uncover LVI-specific biology and to determine whether LVI represents a distinct molecular state rather than a histologic epiphenomenon of tumor aggressiveness.

A molecular definition of LVI has the potential to refine postoperative risk stratification beyond histopathology alone and to identify biologically distinct subsets of LVI-positive disease with divergent clinical behavior. Using data from The Cancer Genome Atlas Prostate Adenocarcinoma Collection (TCGA-PRAD) [22], we aimed to define a confounder-adjusted transcriptomic profile specifically associated with LVI, evaluate its prognostic relevance independent of established clinicopathological factors, and characterize its underlying biological programs through integrated multi-omics analysis.

## 2. Results

### 2.1. Cohort Characteristics and Transcriptomic Profiling

A total of 403 patients with prostate adenocarcinoma from the TCGA-PRAD dataset met the inclusion criteria. Among them, 106 (26.3%) had histopathologic evidence of LVI, and 297 (73.7%) were classified as non-LVI. LVI tumors were significantly associated with higher Gleason scores (χ^2^ test, *p* < 0.001) and advanced pathological T stage (χ^2^ test, *p* < 0.001), confirming the need for adjusted analysis.

**Figure 1:**
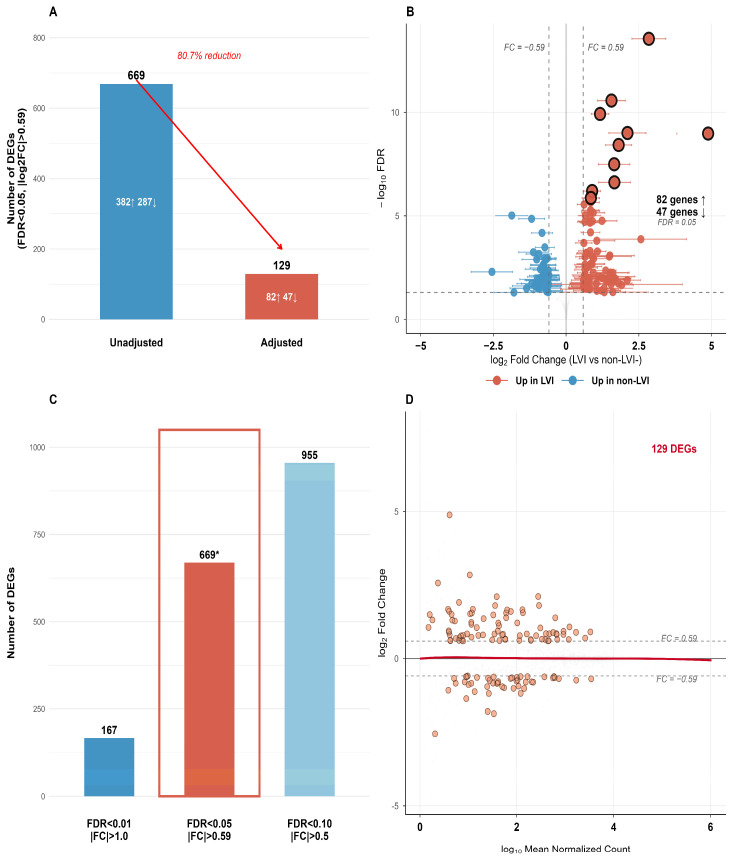
Identification of LVI-specific transcriptional profile in prostate adenocarcinoma. (**A**) Reduction in DEG count after adjusting for Gleason score and pathological T stage. Numbers above bars indicate total DEGs; numbers within bars show genes upregulated (↑) and downregulated (↓) in LVI tumors. The 80.7% reduction indicates substantial confounding by tumor aggressiveness features. (**B**) Volcano plot displaying 129 LVI-associated genes identified after multivariable adjustment. Error bars represent 95% confidence intervals. Red points: 82 genes upregulated in LVI tumors. Blue points: 47 genes downregulated in LVI tumors. Larger circled points represent the ten most differentially expressed genes. Dotted vertical lines indicate |log_2_FC| > 0.59 threshold; dotted horizontal line indicates FDR < 0.05 threshold. (**C**) Sensitivity analysis showing the number of differentially expressed genes (DEGs) identified using different statistical thresholds. The outlined bar represents the primary threshold used in this study (false discovery rate [FDR] < 0.05, absolute log_2_ fold-change [|log_2_FC|] > 0.59). (**D**) Mean-average (MA) plot showing the relationship between mean expression (log_10_ normalized counts) and fold change (log_2_FC). Orange points represent the 129 LVI-associated DEGs. Red line indicates loess regression curve. Dashed horizontal lines represent |log_2_FC| > 0.59 thresholds. *Primary threshold used in this study (FDR < 0.05, |log2FC| > 0.59).

Initial unadjusted differential expression analysis identified 669 genes differentially expressed between LVI and non-LVI groups (FDR < 0.05, |log_2_FC| > 0.59). After multivariable adjustment for Gleason score and pathological T stage using DESeq2, only 129 genes remained independently associated with LVI status, representing an 80.7% reduction (Figure 1A). This substantial decrease indicates that the majority of the transcriptional differences initially attributed to LVI are confounded by underlying tumor aggressiveness features.

The 129 adjusted differentially expressed genes (DEGs) comprised 82 genes upregulated in LVI tumors and 47 genes upregulated in non-LVI tumors (Supplementary Table S1). All exhibited biologically meaningful effect sizes (mean |log_2_FC| = 0.999, range: 0.59–4.87), with confidence intervals excluding the null value for all 129 genes (Figure 1B). Sensitivity analyses using alternative statistical thresholds on unadjusted data demonstrated a 5.7-fold variation in DEG count, ranging from 167 DEGs (FDR < 0.01, |log_2_FC| > 1.0) to 955 DEGs (FDR < 0.10, |log_2_FC| > 0.5) (Figure 1C), confirming that our primary threshold provided a balanced result. The relationship between mean expression and fold change for these genes is shown in Figure 1D.

The 129 adjusted DEGs represent a refined, LVI-specific transcriptional profile suitable for downstream pathway analysis.

### 2.2. The LVI Transcriptional Profile Is Independent of Clinical Confounders and Tumor Microenvironment

To identify LVI-specific transcriptional changes independent of established prognostic factors, we performed multivariable differential expression analysis using DESeq2. Initial univariate comparison revealed 669 DEGs between LVI and non-LVI tumors (FDR < 0.05, |log_2_FC| > 0.59). However, after adjusting for Gleason grade group (≤7 vs. ≥8) and pathological T stage (T2 vs. T3-T4), only 129 genes (82 upregulated and 47 downregulated) retained significance, representing an 80.7% reduction. This substantial attenuation indicates that the majority of apparent LVI-associated expression differences are confounded by tumor grade and local invasion.

**Figure 2:**
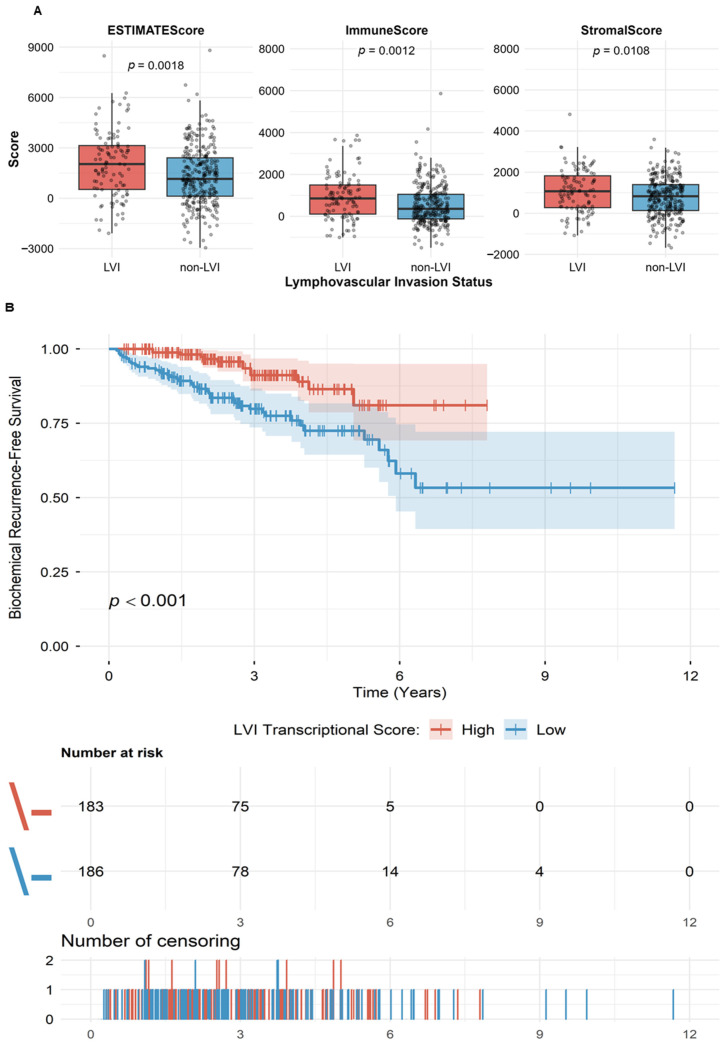
Association of LVI transcriptional profile with tumor microenvironment composition and patient prognosis. (**A**) Comparison of tumor microenvironment scores between LVI and non-LVI prostate tumors using the ESTIMATE algorithm. Boxplots show median (center line), interquartile range (box), and data distribution (points). Statistical significance was assessed using the Wilcoxon rank-sum test. (**B**) Kaplan–Meier analysis of biochemical recurrence-free survival stratified by LVI transcriptional score. Patients were dichotomized at the median score for visualization purposes. High LVI transcriptional score is associated with significantly improved recurrence-free survival compared to low score (log-rank *p* < 0.001). Kaplan–Meier curves with 95% confidence intervals (shaded areas) and censoring indicators (vertical ticks). The lower panel shows the number of patients at risk during follow-up at 0, 3, 6, 9, and 12 years: Red line (High score): n = 183 at baseline → 75 at 3y → 5 at 6y → 0 at 9–12y; Blue line (Low score): n = 186 at baseline → 78 at 3y → 14 at 6y → 4 at 9y → 0 at 12y. Numbers decline due to events (recurrence) and censoring. The lower panel (Number of censoring) shows the count of censored patients at each time point. Note that all statistical analyses (Cox regression models) used the continuous transcriptional score to avoid loss of statistical power; median split is presented here solely for visual representation.

Assessment of tumor microenvironment composition using the ESTIMATE algorithm revealed that LVI tumors exhibited significantly elevated stromal and immune cell content compared to non-LVI tumors (ImmuneScore: 937.9 ± 1102.6 vs. 542.2 ± 937.9, *p* = 0.0012; StromalScore: 1080.8 ± 1054.8 vs. 783.2 ± 916.0, *p* = 0.0108). To determine whether our 129-gene transcriptional profile was independent of these microenvironmental differences, we performed multivariable logistic regression for each gene, additionally adjusting for surgical margin status and tumor purity (ESTIMATE TumorPurity score) (Figure 2A).

Of the 129 genes, 41 (31.8%) remained significantly associated with LVI status after false discovery rate correction (FDR q < 0.05). This gene set exhibited bidirectional effects, with a majority of genes associated with increased LVI risk (odds ratio [OR] > 1) and a substantial subset demonstrating protective effects (OR < 1). The strongest associations included both risk-enhancing genes (e.g., DGKK: OR = 3.72, 95% CI: 1.70–8.15, FDR q = 0.013) and protective genes (e.g., RS1: OR = 0.19, 95% CI: 0.07–0.50, FDR q = 0.013).

To evaluate the prognostic relevance of this refined transcriptional profile, we derived a continuous composite score using principal component analysis (PC1, explaining 13.7% of the variance). In univariable analysis, the LVI transcriptional score was strongly associated with biochemical recurrence-free survival (HR = 0.859, 95% CI: 0.801–0.921, *p* = 1.97 × 10^−5^) (Figure 2B, Table 1).

In multivariable Cox proportional hazards models adjusting for Gleason grade group, pathological T stage, surgical margin status, and tumor purity, the LVI transcriptional score remained independently associated with a reduced risk of biochemical recurrence (adjusted HR = 0.911 per unit score increase, 95% CI: 0.835–0.993, *p* = 0.0334). Each unit increase in transcriptional score corresponded to an approximately 9% reduction in recurrence risk independent of all clinical covariates. To facilitate clinical interpretation, a one-standard-deviation increase in the transcriptional score was associated with a 15% reduction in recurrence risk (HR per 1-SD = 0.85, 95% CI: 0.74–0.97) (Figure 2B, Table 1).

Notably, while the Gleason score (AHR = 2.238, 95% CI: 1.562–3.206, *p* = 1.14 × 10^−5^) and tumor purity (AHR = 0.00016, 95% CI: 2.8 × 10^−6^–0.009, *p* = 2.40 × 10^−5^) showed strong associations with recurrence, the LVI transcriptional profile provided additional independent prognostic information. Bootstrap validation (1000 iterations) confirmed the robustness of this association (bias-corrected HR = 0.916, optimism-corrected concordance index = 0.805).

Collectively, these analyses identify a core 129-gene transcriptional profile associated with LVI in prostate cancer, approximately one-third of which remains significant after comprehensive adjustment for clinicopathological confounders and tumor microenvironment composition. This transcriptional profile not only distinguishes LVI tumors but also provides independent prognostic information beyond conventional risk factors, with higher transcriptional scores predicting a reduced risk of biochemical recurrence.

### 2.3. Network Architecture and Hub Gene Identification

**Figure 3:**
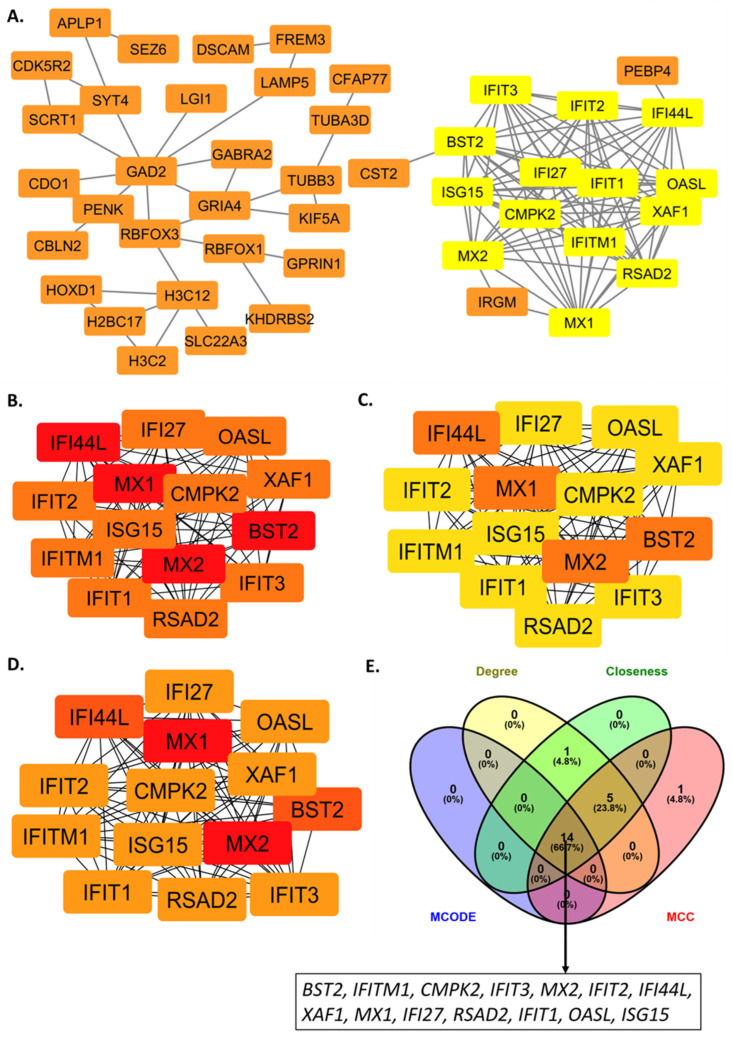
Protein–protein interaction (PPI) network analysis of differentially expressed genes (DEGs) between LVI and non-LVI prostate cancer groups. Each node represents a protein encoded by a DEG, and each edge (lines connecting the nodes) represents a predicted protein–protein interaction. The most significant module within the PPI network, identified using the MCODE Cytoscape (v3.10.3), (yellow) (**A**). Top-ranked hub genes identified using CytoHubba ranking algorithms: degree (**B**), closeness (**C**), and MCC (**D**). Venn diagram showing the intersection of hub genes identified by MCODE and all three CytoHubba algorithms, yielding 14 overlapping hub genes (**E**).

To elucidate the functional architecture underlying the 129 LVI-associated genes, we constructed a protein–protein interaction network (Figure 3). The highest-scoring module (Cluster 1: MCODE score = 14.000) was selected for further characterization, as it represented the most densely interconnected functional unit within the LVI-associated molecular network. This module comprised 14 densely interconnected nodes with 91 edges. Network topological analysis revealed exceptional interconnectivity within Cluster 1, with a network density of 1.0 (indicating that all possible pairwise interactions were present) and an average degree of 13 connections per gene (the maximum possible in a 14-gene module). This perfect connectivity, coupled with a clustering coefficient of 1.0 (indicating perfect local connectivity), confirms the module’s status as a tightly integrated functional unit rather than a spurious association.

Independent hub gene identification using CytoHubba, with multiple centrality algorithms (MCC, Degree, and Closeness), converged on a set of high-confidence hub genes. All 14 genes from MCODE Cluster 1 were consistently ranked as top hubs across all three centrality measures (*BST2*, *IFITM1*, *CMPK2*, *IFIT3*, *MX2*, *IFIT2*, *IFI44L*, *XAF1*, *MX1*, *IFI27*, *RSAD2*, *IFIT1*, *OASL*, and *ISG15*) (Figure 3).

### 2.4. Multi-Omics Characterization of the Interferon-Response Module

**Figure 4:**
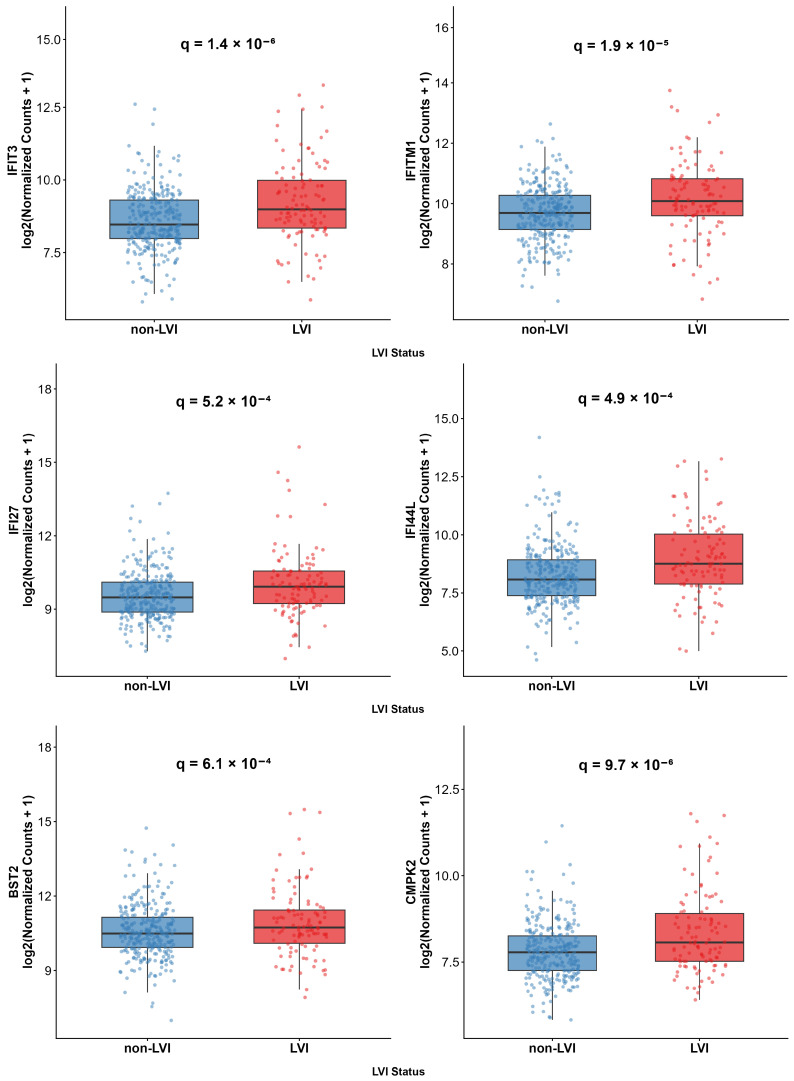

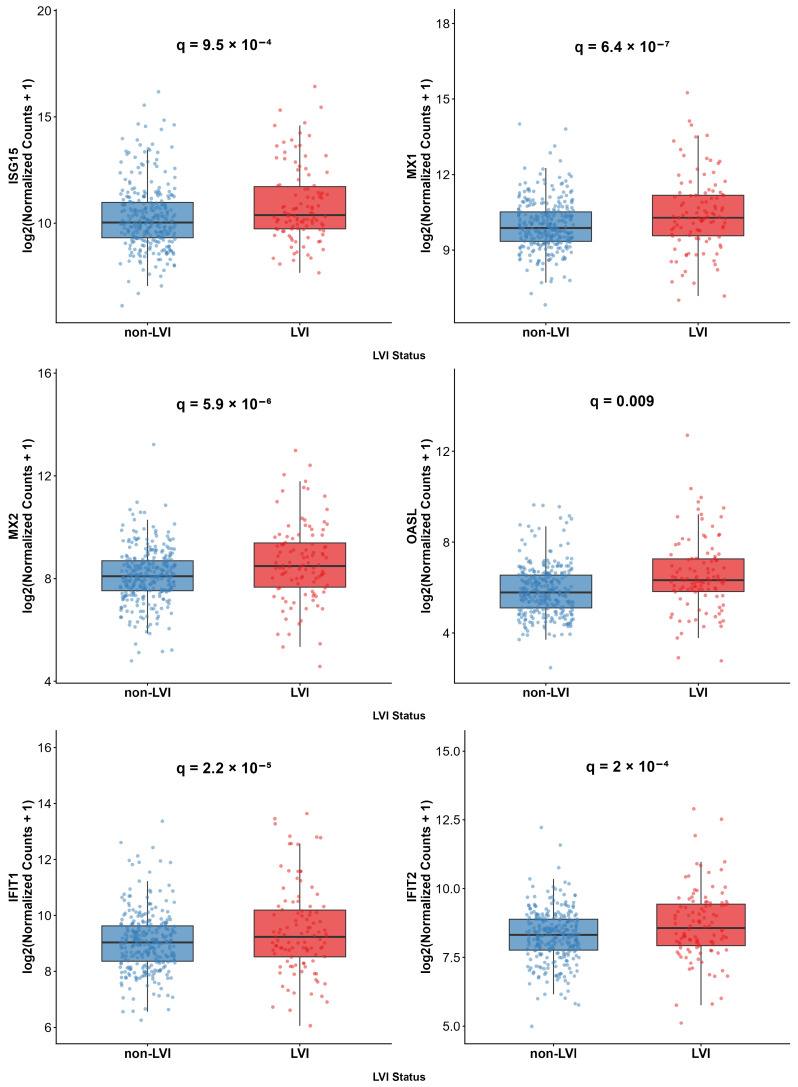
Expression of 14 interferon-response genes in LVI (red) versus non-LVI (blue) prostate tumors. Box plots show log_2_(normalized counts + 1) with individual samples as points. q-values indicate FDR-adjusted significance from DESeq2 analysis adjusted for Gleason score and pathological T stage. Abbreviations: LVI, Lymphovascular invasion.

Expression analysis validated consistent upregulation of all 14 interferon-response genes in LVI tumors (Figure 4). *OASL* exhibited the strongest differential expression (log_2_FC = 1.21, q = 9.28 × 10^−3^), followed by *IFI27* (log_2_FC = 1.07, q = 5.25 × 10^−4^) and *ISG15* (log_2_FC = 0.91, q = 9.46 × 10^−4^). Core antiviral effectors *MX1* (log_2_FC = 0.89, q = 6.40 × 10^−7^) and *MX2* (log_2_FC = 0.84, q = 5.88 × 10^−6^) showed the most significant upregulation, while interferon-induced proteins *IFIT3* (log_2_FC = 0.85, q = 1.38 × 10^−6^), *RSAD2* (log_2_FC = 0.85, q = 2.25 × 10^−6^), and *IFIT1* (log_2_FC = 0.82, q = 2.15 × 10^−5^) demonstrated robust activation of the interferon-response program (Figure 4).

The 14-gene signature showed no significant differential expression in the GSE220095 nodal metastasis cohort (all FDR > 0.78; Supplementary Table S2), while TCGA-PRAD analysis confirmed a strong association between LVI and nodal status (Supplementary Table S3).

Analysis of somatic alterations revealed consistently low mutation frequencies across all 14 interferon-response genes, with no statistically significant differences between LVI and non-LVI tumors after FDR correction (q > 0.05 for all genes). Mutation frequencies ranged from 0% (*IFITM1*) to 14.8% (*MX1*), with the highest frequencies observed for *MX1* and *MX2*. Interestingly, both *MX1* and *MX2* showed nominally higher mutation frequencies in non-LVI tumors (14.8% and 14.4% vs. 9.4% in LVI), though these differences did not reach statistical significance (q = 0.905 and 0.925, respectively). The overall absence of significant genomic alterations supports that transcriptional activation occurs through regulatory rather than structural genomic mechanisms.

**Figure 5:**
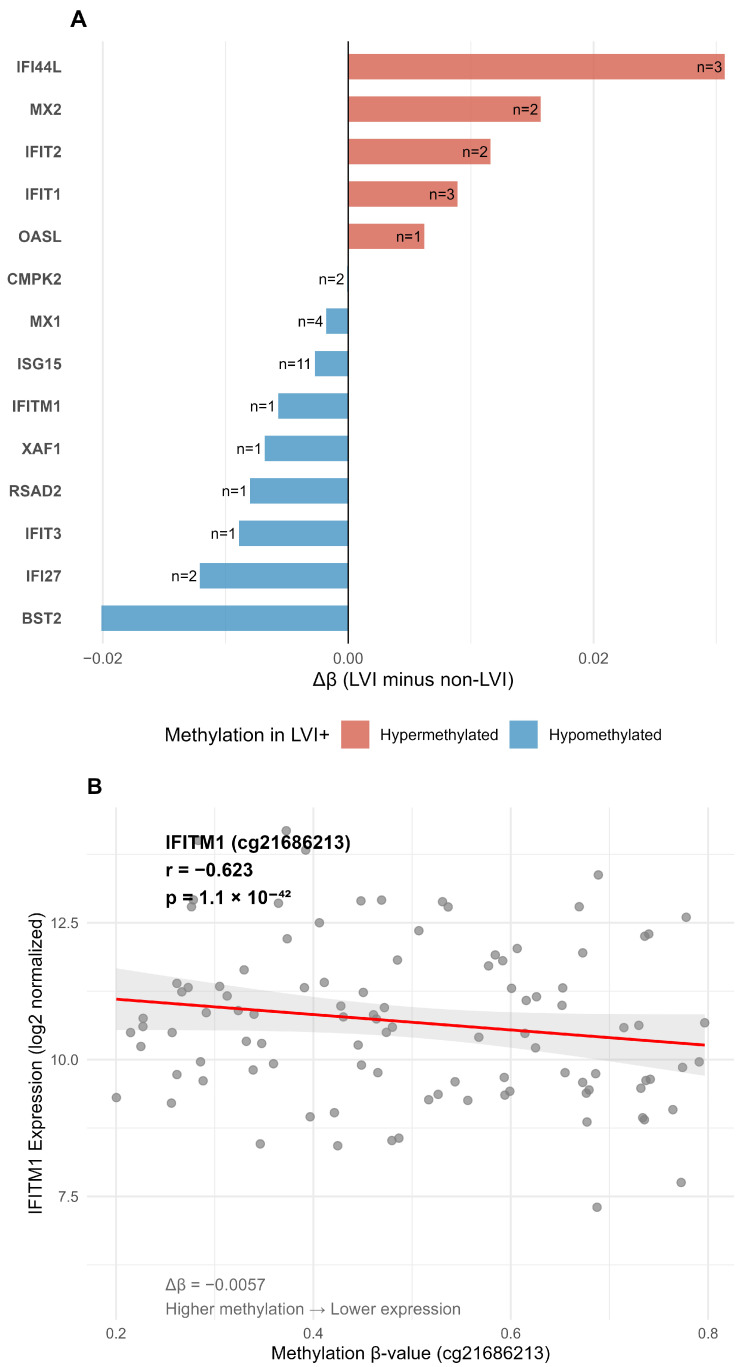
Promoter methylation analysis of interferon-response hub genes. (**A**) Methylation differences (Δβ = LVI minus non-LVI) for all 14 interferon-response hub genes; 34 of 196 CpG probes (17.3%) showed significant differential methylation after FDR correction (q < 0.05). Δβ values ranged from −0.044 to +0.094 (mean absolute Δβ = 0.014). Positive Δβ values (red) indicate hypermethylation in LVI tumors; negative values (blue) indicate hypomethylation. Numbers indicate probes with FDR < 0.05 per gene. (**B**) Methylation–expression correlation for *IFITM1* (probe cg21686213). Higher promoter methylation correlates with lower gene expression (Pearson r = −0.623, FDR = 1.1 × 10^−42^). Δβ = −0.0057 indicates slight hypomethylation in LVI tumors. Red line indicates linear regression fit with 95% confidence interval (shaded area).

Promoter methylation analysis of 196 CpG probes mapping to the 14 interferon-response genes revealed differential methylation in LVI tumors, with 34 probes (17.3%) showing significant differences after FDR correction (q < 0.05) (Figure 5A). Δβ values ranged from −0.044 to +0.094, with mean absolute Δβ = 0.014, indicating subtle promoter methylation changes. The most pronounced differences were observed at cg03607951 (*IFI44L* promoter, Δβ = +0.094, FDR = 7.3 × 10^−4^) and cg07589034 (*ISG15* promoter, Δβ = −0.009, FDR = 3.0 × 10^−3^).

Methylation–expression correlation analysis across 403 matched samples revealed strong negative correlations for multiple interferon genes. The strongest association was observed for *IFITM1* (cg21686213, r = −0.623, FDR = 1.1 × 10^−42^) (Figure 5B), followed by *ISG15* (multiple probes, r ≈ −0.55, FDR < 10^−31^). Nineteen CpG probes exhibited both significant differential methylation between LVI groups and significant negative correlations with expression (FDR < 0.05), supporting functional promoter regulation of interferon-response genes despite quantitatively modest methylation changes.

Protein-level analysis using reverse-phase protein array (RPPA) data from The Cancer Proteome Atlas revealed limited availability of antibodies for the 14 interferon-stimulated genes identified in our transcriptional score. Among available interferon pathway components, only STAT5ALPHA showed significant differential protein expression (fold-change = 0.497, FDR = 3.53 × 10^−3^), representing a 2-fold reduction in LVI tumors. Other key interferon regulators including IRF1 (fold-change = 1.138, FDR = 0.340), IRF3 (fold-change = 0.892, FDR = 0.277), and STAT3 (fold-change = 1.214, FDR = 0.457) showed no significant protein-level differences. This selective protein-level alteration of STAT5ALPHA—a transcription factor that mediates interferon-γ signaling—contrasts with the broad transcriptional upregulation of downstream interferon-stimulated genes, suggesting a disconnected regulatory hierarchy in which transcriptional activation of interferon responses occurs without proportional changes in upstream signaling proteins.

### 2.5. Functional Enrichment Analysis Identifies Interferon Signaling Activation

**Figure 6:**
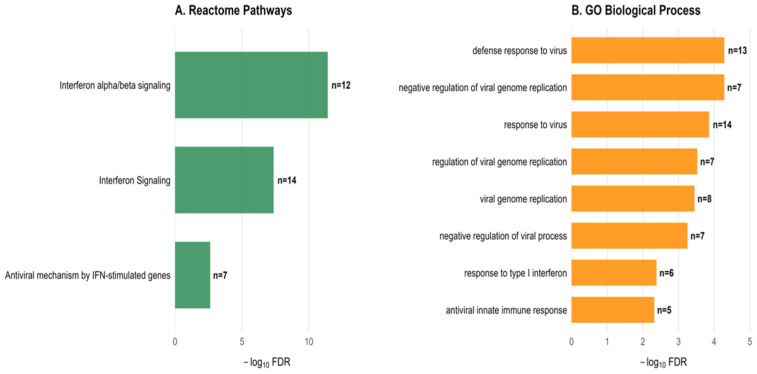

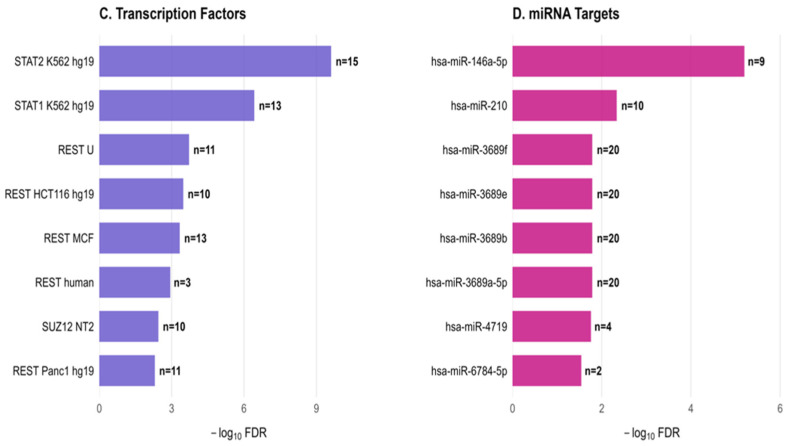
Comprehensive enrichment analysis of LVI-associated transcriptional profile. (**A**) Reactome pathways showing enrichment of interferon signaling in LVI-associated genes. Interferon alpha/beta signaling was the top pathway (FDR = 3.9 × 10^−12^). (**B**) GO Biological Process terms highlighting viral defense mechanisms. (**C**) Transcription factor enrichment identifying STAT2 and STAT1 as key regulators of the interferon response. (**D**) miRNA target analysis revealing hsa-miR-146a-5p as the most significant miRNA targeting LVI-associated genes. Bar labels indicate gene counts (Bar labels indicate gene counts (n values shown on each bar) n=). All results FDR < 0.05.

Enrichment analysis revealed a robust interferon-response program in LVI-associated genes of prostate cancer. Reactome pathway analysis identified “Interferon alpha/beta signaling” as the most significantly enriched pathway (FDR = 3.9 × 10^−12^, 12 genes), indicating the activation of innate immune mechanisms in LVI tumors (Figure 6A). GO Biological Process analysis showed enrichment of viral defense pathways, including “defense response to virus” (FDR = 5.2 × 10^−5^, 13 genes) and “negative regulation of viral genome replication” (FDR = 5.2 × 10^−5^, 7 genes), suggesting molecular mimicry between viral response and LVI biology (Figure 6B). Transcription factor analysis identified STAT2 (*p* = 2.4 × 10^−10^) and STAT1 (*p* = 3.7 × 10^−7^) as key regulators of this transcriptional profile (Figure 6C). In prostate cancer, STAT1/STAT2 signaling has been implicated in immunosurveillance and treatment resistance. miRNA target analysis revealed hsa-miR-146a-5p as the top enriched miRNA (*p* = 6.3 × 10^−6^, 9 targets) (Figure 6D). This miRNA regulates inflammatory responses and has been associated with prostate cancer progression. The enrichment of interferon-response pathways in LVI-associated genes suggests that LVI in prostate cancer may involve activation of immune evasion mechanisms, potentially explaining the aggressive phenotype of LVI tumors.

## 3. Discussion

This study identifies a coherent interferon-response transcriptional program that specifically defines LVI in prostate cancer, after rigorous adjustment for the pronounced confounding effects of Gleason grade and pathological stage. By employing multivariable models that also accounted for tumor microenvironment composition, we isolated 129 genes independently associated with LVI. Network analysis distilled this gene set into a core 14-gene interferon-response module. Critically, a continuous score derived from this transcriptional profile predicted biochemical recurrence-free survival independently of all standard clinicopathological factors, thereby capturing the biologically relevant aspects of LVI more precisely than binary pathological assessment.

The robust enrichment of interferon-alpha/beta signaling pathways represents a significant shift from the classical view of LVI as a consequence of proliferative or epithelial–mesenchymal transition programs [23,24,25,26,27]. While interferon signaling is central to antiviral defense, its role in cancer exhibits context-dependent duality [28,29,30]. We propose a mechanistic model in which chronic interferon stimulation in LVI tumors creates an “inflamed yet permissive” microenvironment conducive to lymphatic dissemination, potentially mediated through interferon-induced chemotaxis, enhanced endothelial adhesion, and the recruitment of pro-metastatic immune cells. This model aligns with our ESTIMATE analysis, showing significantly greater immune-stromal infiltration in LVI tumors.

Recent work has demonstrated that loss of tumor-intrinsic type I interferon signaling characterizes proliferative prostate cancer cells in the bone metastatic niche, where it facilitates immune evasion and metastatic outgrowth [31]. At first glance, this observation may appear discordant with our finding of heightened interferon signaling in LVI-positive primary tumors. However, these findings likely reflect distinct biological states occurring at different stages of disease progression. While suppression of tumor-intrinsic interferon signaling promotes proliferative expansion at metastatic sites, activation of interferon responses in LVI-positive tumors may represent an invasion-competent but non-proliferative state, associated with vascular interaction, cellular stress, and immune engagement during early dissemination. Consistent with this model, interferon activation in LVI-positive tumors may restrain proliferative programs while permitting dissemination, thereby uncoupling invasion from overt tumor growth.

Within this context, the protective prognostic association of our interferon transcriptional profile (adjusted HR = 0.911) presents an apparent paradox but aligns with this duality. A robust interferon response may signify effective immune recognition and an inflamed but controlled state where LVI occurs under active immune surveillance, resulting in better outcomes [32,33]. Conversely, LVI tumors with a weak or absent interferon transcriptional profile might represent an immune-escape phenotype, where invasion proceeds unimpeded, leading to a worse prognosis [34,35]. Thus, our transcriptional profile does not contradict the adverse nature of LVI but rather reveals a prognostically favorable molecular subtype within it, characterized by competent immune engagement [36].

Integrated multi-omics analysis revealed that this transcriptional program is supported by complementary regulatory layers. We observed significant promoter methylation changes, accompanied by strong negative correlations with expression (e.g., *IFITM1*), supporting functional epigenetic modulation [37]. The absence of significant somatic alterations, however, underscores a primary transcriptional/epigenetic dysregulation. The discordance between mRNA and available protein levels further suggests rapid post-transcriptional control. These layers converge to depict a tightly regulated, yet activated, interferon-response state [38,39].

The specificity of the LVI-associated interferon signature was further examined in an independent lymph node metastasis cohort (GSE220095) [40,41]. None of the 14 interferon-response genes showed significant differential expression after multiple testing correction (all FDR > 0.78), confirming that our signature is specific to LVI as a histopathological entity rather than a surrogate for nodal involvement. This finding aligns with the clinical observation that LVI and nodal status, while correlated, are not synonymous—LVI represents the local step of vascular invasion, whereas nodal metastasis requires additional capabilities for colonization. This divergence aligns with recent work by Bustos et al. [41], who identified a four-gene signature (CHRNA2, NPR3, VGLL3, and PAH) for lymph node metastasis with no overlap with our interferon module. The absence of common genes reflects distinct biology: nodal metastasis signatures identified in other studies often include genes involved in ECM remodeling and AR signaling, while LVI-specific programs are dominated by interferon response and immune engagement. These complementary findings underscore that LVI and nodal metastasis represent distinct steps in the metastatic cascade, each with unique transcriptional hallmarks. Notably, in our TCGA-PRAD cohort, LVI showed a strong association with nodal involvement: 48% of LVI-positive patients had lymph node metastases compared to only 5% of LVI-negative patients (*p* < 0.001), and 80% of node-positive patients were LVI-positive. This confirms that while LVI is a major risk factor for nodal spread, our 14-gene signature specifically captures the LVI event itself rather than its downstream consequence.

Clinically, the continuous LVI-transcriptional score provides independent prognostic value, with each unit increase associated with an approximately 9% reduction in recurrence risk. The fact that histological LVI status showed attenuated prognostic significance when the molecular profile was included in models indicates that this molecular assay more accurately captures the biological continuum of LVI than histological dichotomization [42]. This molecular refinement could improve risk stratification as demonstrated by bootstrap validation, confirming model robustness, potentially identifying patients with a molecular LVI phenotype.

Our findings align with and extend recent evidence implicating interferon signaling in prostate cancer progression [31,43,44]. This study complements prior work by specifically linking a robust interferon-expression profile to the key histopathological feature of LVI, employing rigorous confounder-adjustment to isolate the LVI-specific signal, and revealing its associated epigenetic regulation. This positions our work as a focused investigation solving a specific clinical–molecular problem.

Our study has limitations that chart a clear roadmap for future research. The retrospective design necessitates prospective validation in independent cohorts. Although we adjusted for the dominant clinicopathologic confounders and tumor purity, additional biological and clinical factors may influence tumor gene expression, and residual confounding cannot be fully excluded in a discovery-phase analysis. Most importantly, the mechanistic hypotheses generated here require functional validation. Future work should: (1) validate the 14-gene signature in independent cohorts with histopathologically confirmed LVI status, as our attempt in GSE220095 confirmed that the signature is specific to LVI rather than nodal involvement; (2) test the functional role of core interferon genes (e.g., ISG15 and MX1) in models of lymphatic invasion; (3) explore the profile’s predictive value for response to interferon-pathway modulators; and (4) conduct comparative studies to determine how this LVI-specific signature integrates with existing nodal metastasis signatures.

In conclusion, through confounder-adjusted multi-omics analysis, we redefine LVI in prostate cancer as a molecular entity characterized by the activation of the interferon-response pathway. The identified transcriptional profile provides a quantifiable molecular tool that refines prognostic stratification beyond histology, opening new avenues for biologically informed therapy targeting the interferon pathway in aggressive disease.

## 4. Materials and Methods

### 4.1. Study Cohort and Data Acquisition

**Figure 7:**
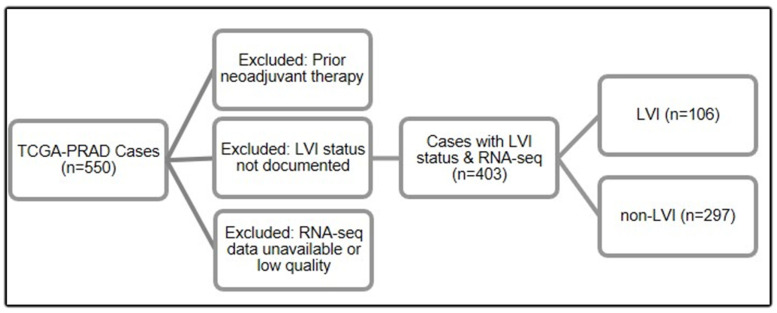
Flow diagram of TCGA-PRAD cohort selection.

In this retrospective cohort study, we analyzed primary prostate adenocarcinoma samples from the TCGA-PRAD project. We initially identified 550 patients in TCGA-PRAD. After applying sequential filters, the final analytical cohort comprised 403 patients who met all inclusion criteria: (1) treatment with radical prostatectomy without prior neoadjuvant therapy, (2) definitive LVI status documented in pathology reports, and (3) available high-quality RNA-seq data (Figure 7). The data generation and initial curation for the TCGA-PRAD cohort have been described previously [22].

LVI status was obtained from the clinicopathologic annotations provided by the TCGA-PRAD project. LVI was abstracted from original histopathology reports at the contributing institutions and recorded as a binary variable (present vs. absent), consistent with standard pathological reporting. This approach parallels the collection of other pathological features in TCGA, including Gleason score, pathological T stage, surgical margin status, and lymph node involvement. In this cohort, LVI is reported as a combined pathological finding (tumor cells within endothelial-lined spaces) without distinction between lymphatic (L1) and vascular (V1) invasion, reflecting standard clinical reporting practices. Cases were classified as LVI based on this designation.

RNA-seq raw count matrices (STAR—Counts workflow) were downloaded from the NCI Genomic Data Commons (GDC) Data Portal. Corresponding clinicopathological data were extracted from TCGA clinical files. Data completeness for key covariates was high: LVI status (100%), Gleason score (100%), pathological T stage (100%), surgical margin status (99.0%), nodal status (97.3%), preoperative prostate-specific antigen (PSA) (96.5%), and age (93.8%). For multivariable models, complete-case analysis was employed, with exact sample sizes reported per model (e.g., n = 369 for Cox regression with 54 events; n = 347 for full logistic regression).

Statistical power for the primary LVI comparison was estimated a priori using the pwr package in R, assuming a two-sided α = 0.05, effect size d = 0.5 (Cohen’s medium effect), and the anticipated unequal group distribution based on typical LVI prevalence (≈25% positive). This calculation yielded >80% power for our final cohort (106 LVI vs. 297 non-LVI).

The stepwise selection of patients from the TCGA-PRAD cohort is illustrated in the flow diagram above (Figure 7). A total of 550 patients with primary prostate adenocarcinoma were initially identified. Patients were sequentially excluded if they had received neoadjuvant therapy prior to radical prostatectomy, lacked definitive LVI status in pathology reports, or had missing or inadequate RNA-sequencing data. After application of all inclusion and exclusion criteria, the final analytical cohort comprised 403 patients, including 106 LVI and 297 non-LVI cases.

### 4.2. Differential Gene Expression Analysis

Gene expression analysis was performed using R (v4.4.2) and the DESeq2 package (v1.40.0) [45]. Raw RNA-seq count data from TCGA-PRAD samples were downloaded from the GDC portal. Protein-coding genes were selected, and low-count genes were filtered (≥10 reads in at least 5 samples), retaining 17,547 genes for analysis.

Differential expression between LVI and non-LVI groups was assessed using negative binomial generalized linear models in DESeq2. To isolate LVI-specific signals from tumor aggressiveness confounders, we employed a staged modeling strategy: (1) unadjusted (LVI only), (2) adjusted for Gleason score (binary: ≤7 vs. ≥8) and pathological T stage (T2 vs. T3/T4), (3) additionally adjusted for surgical margin status, and (4) fully adjusted including preoperative PSA (log-transformed) and patient age. Missing data in clinical covariates (PSA: 3.5%, age: 6.2%) were handled using complete-case analysis for each model.

DEGs were defined using FDR < 0.05 (Benjamini–Hochberg correction) and an absolute log2 fold-change > 0.59 (=1.5-fold change). This threshold was chosen to balance statistical stringency with biological relevance, exceeding typical technical variation while capturing potentially important differences in expression. Log2 fold changes were stabilized using apeglm shrinkage to reduce noise from low-count genes [46]. Sensitivity analyses confirmed the robustness of our findings across alternative thresholds (FDR: 0.01, 0.05, 0.10; |log_2_FC|: 0.5, 0.59, 1.0).

External validation of the 14-gene signature in the GSE220095 cohort (pN0, n = 146; pN1, n = 22) was performed using an identical DESeq2 protocol.

### 4.3. Tumor Purity Estimation and Microenvironment Analysis

Tumor purity was estimated using the ESTIMATE algorithm v1.0.13 based on gene expression profiles. Stromal, immune, and ESTIMATE scores were calculated for each sample, with tumor purity derived using the formula: purity = cos (0.6049872018 + 0.0001467884 × ESTIMATE score) [47].

### 4.4. Multivariable Regression Analyses

#### 4.4.1. Logistic Regression for LVI Association

To identify LVI-associated genes independent of clinicopathological confounders, multivariable logistic regression models were constructed for the 129 LVI-associated genes. Models were adjusted for Gleason grade group (≤7 vs. ≥8), pathologic T stage (T2 vs. T3), surgical margin status (negative vs. positive), and tumor purity (continuous). Gene expression values (VST-normalized counts) were modeled as continuous variables. Odds ratios (OR) with 95% confidence intervals (CI) were calculated, and *p*-values were adjusted for multiple testing using the Benjamini–Hochberg FDR correction (q < 0.05 threshold).

#### 4.4.2. LVI Transcriptional Score Generation

A composite LVI transcriptional score was generated from the 129-gene expression matrix using principal component analysis (PCA). The first principal component (PC1), which explained 13.7% of total variance, was extracted as the continuous LVI transcriptional score. Variance-stabilized transformed (VST) expression values for the 129 LVI-associated genes were mean-centered and analyzed with PCA using prcomp in R. PC1 was selected, as it captured the strongest coordinated expression pattern specifically associated with LVI status. The resulting score represents a weighted linear combination of the 129 genes, with higher values indicating greater transcriptional alignment with LVI-positive tumors (validated in Results 2.2). In Kaplan–Meier visualization, patients are dichotomized into “High” and “Low” groups based on the median of this continuous score for illustrative purposes only; all formal statistical tests utilize the continuous score.

#### 4.4.3. Cox Proportional Hazards Regression for Survival Analysis

Prognostic significance of the LVI transcriptional score was assessed using Cox proportional hazards regression for biochemical recurrence-free survival, defined as the time from radical prostatectomy to PSA ≥0.2 ng/mL. Multivariable models were adjusted for Gleason grade group, pathologic T stage, surgical margin status, and tumor purity (continuous, scaled per 10% increase). This score was modeled as a continuous variable. Hazard ratios (HR) with 95% CIs were reported. Model assumptions were verified using Schoenfeld residuals (proportional hazards) and Martingale residuals (linearity). Model discrimination was quantified using Harrell’s concordance index (C-index).

#### 4.4.4. Internal Validation via Bootstrap

Model robustness was internally validated using bootstrap optimism correction with 1000 iterations. For each iteration: (1) a bootstrap sample was drawn with replacement from the original data, (2) the Cox model was fitted on the bootstrap sample, (3) model performance (C-index) was evaluated on both bootstrap and original data, and (4) optimism was calculated as the difference. The average optimism across 1000 iterations was subtracted from the original concordance index to obtain the optimism-corrected C-index (0.805). Similarly, bias-corrected hazard ratios were obtained by subtracting the average bootstrap coefficient bias (−0.0059) from the original estimates.

### 4.5. Protein–Protein Interaction Network Analysis and Module Detection

A protein–protein interaction (PPI) network was constructed for the 129 LVI-associated genes identified through multivariable logistic regression. Network reconstruction was performed using the STRING database (version 12.0) within the Cytoscape environment (version 3.10.3) [38,39]. Interactions were retrieved with a medium-confidence score threshold of 0.40, which represents the recommended balance between network specificity (minimizing false positives) and sensitivity (capturing true interactions) for biological network reconstruction.

Module detection was conducted using the Molecular Complex Detection (MCODE) algorithm (version 2.0.0). Parameters were set to default values (degree cutoff = 2, node score cutoff = 0.2, k-core = 2, and max depth = 100), as these have been validated for detecting biologically meaningful protein complexes in cancer networks. The highest-scoring module (MCODE score = 14.0) was selected for further characterization, as it represented the most densely interconnected functional unit within the LVI-associated molecular network. Network topological properties, including density, average degree, and clustering coefficient, were calculated using standard graph theory formulas to quantify module interconnectivity.

Hub gene identification employed a two-tiered strategy to ensure robustness. First, topological centrality within the MCODE-identified module was assessed using the cytoHubba plugin (version 0.1) with three complementary algorithms: maximal clique centrality (MCC), degree, and closeness centrality. Genes consistently ranked among the top candidates across all three metrics were identified as core hub genes.

The background gene set for enrichment analysis was defined as all human protein-coding genes with documented interactions in the STRING database, ensuring appropriate statistical correction for the human interactome context.

### 4.6. Pathway Enrichment Analysis

Functional enrichment analysis was performed on the full set of 129 LVI-associated DEGs using clusterProfiler (v4.10.0) [48]. To ensure appropriate statistical context and avoid circularity, analyses were conducted against the entire human protein-coding genome (n = 19,385 genes) as background.

Pathway databases included ReactomePA and Gene Ontology Biological Processes. Transcription factor enrichment was analyzed using the ChEA, ENCODE, TRRUST, miRTarBase, and TargetScan databases via the enricher function [49]. miRNA target analysis utilized the miRTarBase database through the enrichR package (v3.2) [50]. All enrichment results were FDR-corrected across databases using the Benjamini–Hochberg method (q < 0.05).

The 14-gene interferon-response module identified through network analysis was treated as a secondary, hypothesis-generating finding to address potential circularity concerns, with primary enrichment analysis performed on all 129 DEGs.

## 5. Conclusions

In this integrated multi-omics analysis, we demonstrate that LVI in prostate cancer represents more than a nonspecific marker of tumor aggressiveness. After rigorous adjustment for dominant clinicopathologic confounders, LVI is associated with a distinct transcriptional program characterized by activation of interferon-response pathways, accompanied by coordinated epigenetic regulation. These findings indicate that LVI reflects a specific biological state rather than merely the downstream consequence of high-grade or advanced-stage disease.

Importantly, the derived interferon-response transcriptional profile provides prognostic information independent of standard clinicopathological variables, identifying a subset of LVI-positive tumors with comparatively favorable outcomes. This observation highlights molecular heterogeneity within LVI-positive disease and suggests that histopathologic assessment alone may insufficiently capture biologically and clinically relevant differences among these tumors.

From a translational perspective, these results refine the conceptual framework of LVI as an immunologically active tumor–host interface rather than a purely proliferative or mechanical step in tumor dissemination. By linking LVI to interferon signaling, this work provides a biologically grounded hypothesis for future functional studies and raises the possibility that interferon-related pathways may serve as therapeutic or predictive targets in selected patients with aggressive prostate cancer. Collectively, our findings establish a molecular foundation for improved risk stratification and motivate future validation and mechanistic studies to determine how LVI-associated immune signaling can be leveraged clinically.

## Figures and Tables

**Table 1:** Univariable and Multivariable Cox Proportional Hazards Analyses for Biochemical Recurrence.

	Univariable Analysis		Multivariable Analysis	
	HR (95% CI)	*p*	AHR (95% CI)	*p*
LVI Transcriptional Score (per unit)	0.859 (0.801–0.921)	1.97 × 10^−5^	0.911 (0.835–0.993)	0.0334
Gleason Grade Group				
≤7	Reference	-	Reference	
≥8 vs. ≤7	6.135 (3.285–11.457)	2.89 × 10^−11^	2.238 (1.562–3.206)	1.14 × 10^−5^
Pathologic T Stage				
T2	Reference	-	Reference	-
T3+ vs. T2	1.762 (1.326–2.342)	9.59 × 10^−5^	0.889 (0.622–1.270)	0.5163
Surgical Margin				
Negative	Reference	-	Reference	-
Positive	2.164 (1.481–3.161)	6.53 × 10^−5^	1.478 (0.930–2.349)	0.0985
Tumor Purity (per unit increase)	0.00230 (1.00006–1.086)	0.000879	0.00016 (2.8 × 10^−6^–0.009)	2.40 × 10^−5^

Abbreviations: HR, hazard ratio; AHR, adjusted hazard ratio; CI, confidence interval. Univariable analyses: n = 369, events = 54. Multivariable model: n = 369, events = 54, Concordance (C-index) = 0.809. LVI transcriptional score represents the first principal component of 129 LVI-associated genes. Tumor purity is estimated by the ESTIMATE algorithm.

## Data Availability

The data used in this study are publicly available from The Cancer Genome Atlas (TCGA) via the NCI Genomic Data Commons (GDC) portal (https://portal.gdc.cancer.gov/ (accessed on 1 October 2025)) under the TCGA-PRAD project. The complete analysis code supporting the findings of this study is available from the corresponding author upon reasonable request.

## Supplementary Materials

The following supporting information can be downloaded at: https://www.mdpi.com/article/10.3390/ijms27052167/s1.

## Abbreviations

ADT	Androgen deprivation therapy
AR	Androgen receptor
BP	Biological process
CDK	Cyclin-dependent kinase
CI	Confidence interval
DEG	Differentially expressed gene
EMT	Epithelial–mesenchymal transition
FDR	False discovery rate
GO	Gene ontology
HM450	HumanMethylation450
HR	Hazard ratio
KEGG	Kyoto Encyclopedia of Genes and Genomes
LVI	Lymphovascular invasion
MCODE	Molecular complex detection
MF	Molecular Function
PPI	Protein–protein interaction
PRAD	Prostate adenocarcinoma
PSA	prostate-specific antigen
RNA-Seq	RNA sequencing
RPPA	Reverse-phase protein array
STRING	Search Tool for the Retrieval of Interacting Genes/Proteins
TCGA	The Cancer Genome Atlas
