# Supplementary material for: An Interferon-Response Transcriptomic Signature of Lymphovascular Invasion in Prostate Cancer
Source: Int J Mol Sci. 2026 Feb 25;27(5):2167. doi: 10.3390/ijms27052167 (PMC12984949; doi:10.3390/ijms27052167)
Supplement: Supplementary file 1 [file ijms-27-02167-s001.zip › ijms-3986281-supplementary.pdf]

**Supplementary Table S1**. Confounder-adjusted differentially expressed genes associated with lymphovascular invasion in TCGA-PRAD.						
Gene_Symbol	log2 Fold Change	Fold Change	95% CI Lower	95% CI Upper	FDR	Regulation
CD5L	4.882919	29.50565	3.808174	5.957664	1.07E-09	Up_in_LVI
DGKK	2.843092	7.175562	2.26E+00	3.425543	2.85E-14	Up_in_LVI
GPR101	2.568532	5.932057	9.95E-01	4.141838	1.35E-04	Up_in_LVI
RS1	-2.55097	0.17064	-3.26261	-1.83933	5.07E-03	Up_in_nonLVI
SYT4	2.107842	4.310461	1.4688	2.746884	1.01E-09	Up_in_LVI
ECEL1	2.102613	4.294864	1.65369	2.551535	1.28E-02	Up_in_LVI
SCRT1	1.90866	3.754601	1.509884	2.307436	2.20E-02	Up_in_LVI
WDR72	-1.86899	0.273766	-2.41946	-1.31851	9.68E-06	Up_in_nonLVI
ETV4	1.804149	3.492232	1.354626	2.253673	3.81E-09	Up_in_LVI
SEZ6	1.800946	3.484487	1.335052	2.26684	1.86E-02	Up_in_LVI
VWA5B1	-1.79405	0.288362	-2.47394	-1.11415	4.97E-02	Up_in_nonLVI
TNR	1.675127	3.193475	1.152674	2.197581	1.14E-02	Up_in_LVI
CCDC194	1.673657	3.190223	1.11899	2.228325	1.03E-02	Up_in_LVI
CBLN2	1.658051	3.155899	1.106741	2.209361	2.42E-07	Up_in_LVI
SLC22A10	1.651086	3.140699	1.11213	2.190042	3.25E-08	Up_in_LVI
IRGM	1.605531	3.043078	1.172604	2.038458	4.76E-02	Up_in_LVI
TBX10	1.601531	3.034652	1.080117	2.122945	5.63E-03	Up_in_LVI
PLCXD3	1.557131	2.94268	1.076784	2.037478	2.74E-11	Up_in_LVI
CDK5R2	1.549249	2.926647	1.125842	1.972655	1.17E-02	Up_in_LVI
FGB	1.548901	2.925943	-0.89669	3.994495	2.05E-02	Up_in_LVI
CAMKV	1.519217	2.866355	0.870925	2.16751	5.56E-03	Up_in_LVI
MMD2	1.50195	2.832253	0.663388	2.340512	8.26E-04	Up_in_LVI
ERVV-2	1.495614	2.819841	0.625979	2.365249	8.54E-03	Up_in_LVI
FREM3	1.48231	2.793958	0.72252	2.242101	9.22E-04	Up_in_LVI
PTPRR	1.389062	2.619083	0.900427	1.877697	1.50E-02	Up_in_LVI
CST2	1.385702	2.61299	0.799133	1.972271	1.12E-02	Up_in_LVI
CEACAM7	-1.35995	0.389597	-1.931	-0.78889	3.15E-02	Up_in_nonLVI
TLCD3B	1.35714	2.561768	0.996356	1.717923	4.44E-03	Up_in_LVI
GAD2	1.311558	2.482095	-0.21346	2.83658	4.81E-02	Up_in_LVI
UGT2B10	1.304402	2.469814	0.330798	2.278007	3.16E-02	Up_in_LVI
IGFL2	1.276753	2.42293	0.755556	1.797949	1.06E-02	Up_in_LVI
GRIA4	1.231048	2.347374	0.715917	1.746178	1.75E-05	Up_in_LVI
OASL	1.212156	2.316836	0.905983	1.518328	9.28E-03	Up_in_LVI
LGI1	1.209839	2.313118	0.554264	1.865414	4.02E-02	Up_in_LVI
C6	-1.18632	0.439421	-1.63014	-0.74251	1.39E-05	Up_in_nonLVI
CLDN10	-1.18271	0.440523	-1.69001	-0.67542	1.89E-02	Up_in_nonLVI
CRYBB1	1.164688	2.241847	0.863586	1.46579	1.22E-10	Up_in_LVI

TNMD	-1.12356	0.458959	-1.63081	-0.61631	5.72E-04	Up_in_nonLVI
GABRA2	1.116023	2.167487	0.442606	1.78944	8.60E-03	Up_in_LVI
NKX6-2	-1.07671	0.47411	-1.84485	-0.30857	2.42E-02	Up_in_nonLVI
IFI27	1.073657	2.104761	0.780562	1.366751	5.25E-04	Up_in_LVI
CA5A	1.057334	2.081083	0.251151	1.863517	4.12E-02	Up_in_LVI
HOXD1	1.051854	2.073192	0.430815	1.672893	1.60E-04	Up_in_LVI
CYP4F2	1.040279	2.056626	0.207851	1.872708	1.86E-02	Up_in_LVI
TTR	-1.01584	0.494541	-1.53408	-0.49759	1.89E-02	Up_in_nonLVI
MAB21L1	-1.01232	0.495748	-1.51775	-0.50689	1.27E-03	Up_in_nonLVI
SNTN	-0.99384	0.502138	-1.59247	-0.39522	1.15E-02	Up_in_nonLVI
VNN1	-0.95642	0.515333	-1.26196	-0.65089	2.62E-02	Up_in_nonLVI
MKRN3	-0.95289	0.516595	-1.75109	-0.15469	3.16E-02	Up_in_nonLVI
CYP17A1	0.942646	1.92205	0.449593	1.435699	1.16E-03	Up_in_LVI
PROK1	-0.93778	0.522035	-1.37503	-0.50053	6.97E-04	Up_in_nonLVI
SYNGR3	0.931863	1.907738	0.538456	1.32527	7.10E-06	Up_in_LVI
S100P	0.927162	1.901532	0.386488	1.467836	5.63E-03	Up_in_LVI
ISG15	0.905198	1.872801	0.473116	1.33728	9.46E-04	Up_in_LVI
TAC3	0.901943	1.86858	0.433453	1.370432	1.83E-05	Up_in_LVI
MX1	0.892149	1.855939	0.600511	1.183788	6.40E-07	Up_in_LVI
C1QTNF3	0.884247	1.845801	0.442913	1.325581	2.12E-03	Up_in_LVI
IRX6	-0.88402	0.541857	-1.40846	-0.35957	3.78E-03	Up_in_nonLVI
IFIT3	0.848702	1.800879	0.550792	1.146611	1.38E-06	Up_in_LVI
RSAD2	0.846129	1.797671	0.552763	1.139494	2.25E-06	Up_in_LVI
RPE65	-0.84385	0.557154	-1.41426	-0.27345	1.27E-02	Up_in_nonLVI
MX2	0.843084	1.793881	0.534015	1.152153	5.88E-06	Up_in_LVI
TDGF1	0.839583	1.789532	0.376708	1.302457	2.11E-03	Up_in_LVI
LINGO2	-0.83824	0.559325	-1.40882	-0.26766	1.22E-02	Up_in_nonLVI
FSIP1	0.838185	1.7878	0.551549	1.124821	5.28E-06	Up_in_LVI
CFAP299	-0.83709	0.559771	-1.36623	-0.30796	1.34E-02	Up_in_nonLVI
C16orf54	0.83668	1.785936	0.513716	1.159644	6.29E-05	Up_in_LVI
INSYN2B	-0.83331	0.561241	-1.325	-0.34161	8.86E-03	Up_in_nonLVI
INSYN2A	-0.82799	0.563315	-1.18705	-0.46892	6.68E-05	Up_in_nonLVI
KIF5A	0.8271	1.774115	0.46843	1.18577	9.68E-06	Up_in_LVI
IFIT1	0.82069	1.766251	0.504454	1.136927	2.15E-05	Up_in_LVI
IFI44L	0.817152	1.761924	0.435861	1.198442	4.94E-04	Up_in_LVI
TM4SF20	0.810392	1.753687	0.07471	1.546073	3.15E-02	Up_in_LVI
LRRTM3	0.803966	1.745894	-0.19533	1.803264	3.48E-02	Up_in_LVI
ADAMTS6	0.80285	1.744545	0.457751	1.14795	1.98E-05	Up_in_LVI
DPT	-0.80177	0.573645	-1.24078	-0.36276	3.29E-03	Up_in_nonLVI
PENK	-0.79848	0.574956	-1.26433	-0.33262	7.72E-03	Up_in_nonLVI
LRRC18	-0.79473	0.57645	-1.23058	-0.35889	2.45E-03	Up_in_nonLVI

DPYS	-0.78998	0.578351	-1.37856	-0.20141	2.72E-02	Up_in_nonLVI
DSCAM	0.788526	1.727309	0.330927	1.246125	1.12E-02	Up_in_LVI
IL36RN	0.787289	1.725828	0.128856	1.445721	3.28E-02	Up_in_LVI
RBFOX1	-0.78542	0.580185	-1.21334	-0.3575	2.25E-03	Up_in_nonLVI
FNDC1	0.779591	1.716644	0.331896	1.227286	2.79E-03	Up_in_LVI
APLP1	0.778393	1.715219	0.51151	1.045275	1.50E-02	Up_in_LVI
AC138969.1	0.769375	1.704532	0.286409	1.252342	1.00E-02	Up_in_LVI
IGSF1	-0.76202	0.589671	-1.17958	-0.34445	2.45E-03	Up_in_nonLVI
CFAP221	-0.74183	0.597979	-1.09976	-0.3839	1.12E-03	Up_in_nonLVI
PEBP4	-0.73507	0.600787	-1.13284	-0.33731	4.18E-03	Up_in_nonLVI
CA14	-0.73155	0.602258	-1.05983	-0.40327	3.31E-04	Up_in_nonLVI
H2BC17	0.726171	1.654243	0.363573	1.088769	6.89E-04	Up_in_LVI
FBXO39	0.711512	1.637519	0.340672	1.082352	3.24E-03	Up_in_LVI
BST2	0.696294	1.620338	0.379127	1.013462	6.15E-04	Up_in_LVI
SLC22A3	-0.68802	0.620704	-1.02767	-0.34838	1.27E-03	Up_in_nonLVI
H3C2	0.68286	1.605318	0.112498	1.253221	3.33E-02	Up_in_LVI
PAGE4	-0.68147	0.623531	-1.08046	-0.28247	7.07E-03	Up_in_nonLVI
SLC22A7	-0.67546	0.626135	-1.23297	-0.11795	4.00E-02	Up_in_nonLVI
KY	-0.6751	0.62629	-1.1138	-0.2364	7.98E-03	Up_in_nonLVI
CFAP77	-0.66707	0.629784	-1.19984	-0.13431	4.45E-02	Up_in_nonLVI
LRRTM4	0.666165	1.586849	0.154003	1.178327	1.91E-02	Up_in_LVI
CMPK2	0.660491	1.580621	0.414627	0.906356	9.68E-06	Up_in_LVI
RBFOX3	-0.65847	0.633551	-1.03992	-0.27702	7.25E-03	Up_in_nonLVI
TMEM252	-0.6552	0.63499	-1.08285	-0.22754	1.22E-02	Up_in_nonLVI
CDO1	-0.65073	0.636957	-1.0801	-0.22136	1.22E-02	Up_in_nonLVI
KCNK15	0.648295	1.567315	0.333221	0.963369	9.46E-04	Up_in_LVI
SERPINI1	0.646921	1.565823	0.295437	0.998404	2.29E-03	Up_in_LVI
IFITM1	0.646885	1.565784	0.39859	0.89518	1.95E-05	Up_in_LVI
XAF1	0.64682	1.565713	0.294676	0.998963	4.04E-03	Up_in_LVI
LAMP5	0.646179	1.565018	0.211025	1.081333	7.07E-03	Up_in_LVI
VIT	-0.64394	0.639963	-1.02617	-0.26171	7.07E-03	Up_in_nonLVI
CNGA4	-0.64305	0.640356	-0.94822	-0.33788	1.11E-03	Up_in_nonLVI
KCTD16	0.635591	1.553574	0.186713	1.084469	2.44E-02	Up_in_LVI
C1QL1	-0.62534	0.648267	-1.06701	-0.18367	2.42E-02	Up_in_nonLVI
TUBB3	0.623397	1.540498	0.241834	1.00496	8.08E-03	Up_in_LVI
TUBA3D	-0.62121	0.650125	-1.01238	-0.23004	1.10E-02	Up_in_nonLVI
GDF3	0.620339	1.537237	0.130002	1.110677	1.84E-02	Up_in_LVI
H3C12	0.618486	1.535263	0.17147	1.065501	1.63E-02	Up_in_LVI
GPRIN1	0.618409	1.535181	0.385316	0.851501	2.82E-06	Up_in_LVI
ESM1	0.618089	1.534841	0.102596	1.133583	3.13E-02	Up_in_LVI
STOML3	-0.61684	0.652098	-1.1754	-0.05828	4.81E-02	Up_in_nonLVI

KBTBD13	-0.61568	0.652621	-1.08569	-0.14568	2.31E-02	Up_in_nonLVI
IP6K3	-0.61424	0.653276	-1.06237	-0.16611	1.83E-02	Up_in_nonLVI
PPP1R1A	-0.61374	0.6535	-0.99915	-0.22833	9.67E-03	Up_in_nonLVI
JPH4	-0.61198	0.654298	-0.95543	-0.26853	4.36E-03	Up_in_nonLVI
ECT2L	0.611186	1.527514	0.204167	1.018205	2.80E-02	Up_in_LVI
IFIT2	0.608959	1.525158	0.331189	0.886728	2.03E-04	Up_in_LVI
KHDRBS2	0.607094	1.523188	0.119393	1.094796	2.16E-02	Up_in_LVI
FBXL16	0.596821	1.512381	0.182131	1.011511	1.26E-02	Up_in_LVI
LHX2	0.595891	1.511406	0.096514	1.095269	2.74E-02	Up_in_LVI
LIPJ	-0.59312	0.662908	-0.94543	-0.24081	7.25E-03	Up_in_nonLVI

Supplementary Table S2. Differential Expression Analysis of 14-Gene Interferon Signature in GSE220095 (pN1 vs pN0)

Gene_Symbol	log2 Fold Change	p-value	FDR	Significant (FDR < 0.05)?
BST2	1.08 x 10-5	0.1416	0.782	NO
ISG15	-3.81 x 10-6	0.1591	0.794	NO
IFI44L	-5.87 x 10-6	0.2906	0.852	NO
IFIT1	-4.70 x 10-6	0.3044	0.859	NO
XAF1	1.15 x 10-4	0.3120	0.864	NO
IFIT3	5.90 x 10-5	0.3142	0.865	NO
IFI27	2.37 x 10-6	0.3647	0.880	NO
OASL	2.14 x 10-6	0.4936	0.917	NO
MX2	-3.26 x 10-6	0.4995	0.917	NO
IFIT2	4.07 x 10-6	0.5329	0.925	NO
IFITM1	-2.06 x 10-6	0.6798	0.948	NO
CMPK2	-2.16 x 10-6	0.6809	0.948	NO
MX1	-1.96 x 10-5	0.7235	0.955	NO
RSAD2	-4.43 x 10-7	0.9381	0.990	NO

Analysis Note: All 14 genes showed FDR > 0.78, indicating no significant differential expression between pN1 and pN0 tumors.

**Supplementary Table S3.** TCGA-PRAD Cohort: LVI and Nodal Status Association			
	N0	N1	Total
LVI+	51	47	98
LVI-	228	12	240
Total	279	59	338

$p < 0.001$ (Chi-square test)
